# Development of a nomogram to estimate the risk of community‐acquired pneumonia in adults with acute asthma exacerbations

**DOI:** 10.1111/crj.13706

**Published:** 2023-10-04

**Authors:** Yufan Duan, Dilixiati Nafeisa, Mengyu Lian, Jie Song, Jingjing Yang, Ziliang Hou, Jinxiang Wang

**Affiliations:** ^1^ Department of Pulmonary and Critical Care Medicine, Beijing Luhe Hospital Capital Medical University Beijing China

**Keywords:** asthma exacerbations, clinical features, community‐acquired pneumonia, nomogram

## Abstract

**Objective:**

The aim of this study is to investigate the clinical characteristics of acute asthma exacerbations (AEs) with community‐acquired pneumonia (CAP) in adults and establish a CAP prediction model for hospitalized patients with AEs.

**Methods:**

We retrospectively collected clinical data from 308 patients admitted to Beijing Luhe Hospital, Capital Medical University, for AEs from December 2017 to August 2021. The patients were divided into CAP and non‐CAP groups based on whether they had CAP. We used the Lasso regression technique and multivariate logistic regression analysis to select optimal predictors. We then developed a predictive nomogram based on the optimal predictors. The bootstrap method was used for internal validation. We used the area under the receiver operating characteristic curve (AUC), calibration curve, and decision curve analysis (DCA) to assess the nomogram's discrimination, accuracy, and clinical practicability.

**Results:**

The prevalence of CAP was 21% (65/308) among 308 patients hospitalized for AEs. Independent predictors of CAP in patients hospitalized with an AE (*P* < 0.05) were C‐reactive protein > 10 mg/L, fibrinogen > 4 g/L, leukocytes > 10 × 10^9^/L, fever, use of systemic corticosteroids before admission, and early‐onset asthma. The AUC of the nomogram was 0.813 (95% CI: 0.753–0.872). The concordance index of internal validation was 0.794. The calibration curve was satisfactorily consistent with the diagonal line. The DCA indicated that the nomogram provided a higher clinical net benefit when the threshold probability of patients was 3% to 89%.

**Conclusions:**

The nomogram performed well in predicting the risk of CAP in hospitalized patients with AEs, thereby providing rapid guidance for clinical decision‐making.

## Graphical abstract

Community‐acquired pneumonia (CAP) is a common comorbidity in patients with asthma exacerbations (AEs). Our findings confirm that patients with AEs and CAP have distinct clinical features and a high incidence. Early‐onset asthma, fever, use systemic corticosteroids before admission, and some markers (the increased white blood cell, fibrinogen, and C‐reactive protein [CRP]) were independent predictors of CAP in patients hospitalized for AEs.

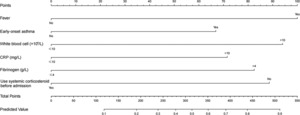

## 1 INTRODUCTION

Asthma is a common chronic inflammatory disease of the airways and affected an estimated 262 million people in 2019, according to the Global Burden of Disease.^1^ The prevalence of asthma among adults over 20 years of age in China is 4.2% or approximately 45.7 million people.^2^ Acute asthma exacerbations (AEs) often lead to emergency medical care, missed work and school, and hospitalization,^3^, ^4^ resulting in a heavy social and economic burden.^5^, ^6^

Community‐acquired pneumonia (CAP) is a common comorbidity in patients with AEs.^7^, ^8^, ^9^, ^10^ However, there are few studies on adults with AEs and CAP. In previous studies on the prognosis of patients hospitalized for AEs, the prevalence of CAP in adult patients of AEs varied widely, from 7% to 30.0%.^9^, ^10^, ^11^, ^12^ Moreover, studies have shown pneumonia to be an independent risk factor for death in patients hospitalized for AEs.^9^, ^13^ No studies describing the clinical features of adults who suffered from AEs with CAP have been reported.

We retrospectively analyzed 308 patients hospitalized for AEs at Beijing Luhe Hospital, Capital Medical University, from December 2017 to August 2021 to promptly identify the high‐risk population of CAP in adults with AEs. We identified some of the clinical characteristics of adult AEs patients with CAP. We developed a prediction model to evaluate the CAP risk in adults with AEs.

## 2 METHODS

### 2.1 Population and study design

We retrospectively analyzed medical records of patients admitted to the Department of Respiratory and Critical Care Medicine, Beijing Luhe Hospital, Capital Medical University, from December 2017 to August 2021. All patients had a discharge diagnosis of AEs. A bronchial asthma diagnosis was made according to the Global Initiative for Asthma (GINA) diagnostic criteria.^14^ AEs were defined according to GINA as the sudden onset of symptoms such as wheezing, shortness of breath, cough, chest tightness, or a sharp worsening of existing symptoms with progressively impaired lung function, usually requiring a change in treatment. The severity of AEs is classified as mild to moderate, severe, and life‐threatening according to the GINA.

The inclusion criteria of the study included the following: age ≥ 18 years and hospitalization for AEs. Exclusion criteria included the following: age < 18 years, a discharge within 24 h of admission or incomplete information, pregnancy, pulmonary vasculitis, human immunodeficiency virus infection, and receiving immunosuppressive therapy.

This study was performed in accordance with the principles of the Declaration of Helsinki. The research was approved by the Ethics Committee of Beijing Luhe Hospital, Capital Medical University (No: 2021‐LHKY‐055‐02). As it was a noninterventional observational study using anonymous data, the ethics committee issued a waiver for informed consent.

### 2.2 Data collection and definitions

Clinical data on these patients were collected anonymously within 24 h of admission from electronic medical records. The data consist of the following six categories: (I) demographic characteristics: age, gender, body mass index (BMI), and smoking; (II) clinical outcomes: length of hospital stay and total costs of hospitalization; (III) clinical characteristics: asthma duration, early‐onset asthma, family history of asthma, hospitalization due to AE in the past year, AEs severity, prehospital maintenance medications, inhaled corticosteroid (ICS) types, and use systemic corticosteroids before admission; (IV) major comorbidities: hypertension, congestive heart failure, coronary heart disease, diabetes mellitus, chronic obstructive pulmonary disease (COPD), allergic rhinitis, stroke, chronic renal disease, gastroesophageal reflux disease, and the calculated Charlson Comorbidity Index score; (V) laboratory examinations: white blood cell, neutrophils, eosinophils, lymphocytes, platelet, neutrophil‐to‐lymphocyte ratio (NLR), platelet‐to‐lymphocyte ratio (PLR), C‐reactive protein (CRP), procalcitonin (PCT), fibrinogen, albumin, and blood urea nitrogen; (VI) primary symptoms and vital signs on admission: fever, respiratory rate, heart rate, and oxygen saturation.

Fever is described as body temperatures >37.3°C. Patients are classified as early‐onset or late‐onset asthma depending on their age at the first presentation of asthma symptoms: ≤40 or >40 years old, respectively. This age cutoff value is based on prior literature.^15^, ^16^, ^17^ A nonsmoker is defined as someone who has never smoked. A current smoker is defined as a regular and occasional smoker, having smoked ≥100 cigarettes during their lifetimes. Former smokers are those who reported having smoked ≥100 cigarettes during their lifetimes but are not smoking during the interview.^18^ The use of systemic corticosteroids before admission is defined as using oral or intravenous corticosteroids for treating an acute AE within 7 days before hospitalization.^19^

### 2.3 Outcome

The outcome indicator was CAP. The patients were divided into CAP and non‐CAP groups based on the Infectious Diseases Society of America/American Thoracic Society (IDSA/ATS) guidelines for CAP in adults.^20^ The primary goals of this study were to understand the clinical features of AEs with CAP in adults and to construct a prediction model of CAP for hospitalized patients with AEs.

### 2.4 Statistical analysis

Multiple imputation was used to handle the missing values in some variables. We used all the predictors and the outcome indicator in the imputation model. Considering the sample size and statistical efficiency, we performed five imputations. We integrated the results of the imputed datasets according to Rubin's rules.^21^ Finally, we used the integrated data to select optimal predictors and develop the prediction model.

We used R4.1.3 software for statistical analysis. All tests were performed using two‐sided tests, with *P* < 0.05 indicating that the difference was statistically significant. We described continuous variables conforming to a normal distribution as mean ± standard deviation and those meeting a nonnormal distribution as the median and interquartile range (IQR). Categorical data were presented as the frequency and the percentage. We used the Mann–Whitney *U*‐test, Fisher's exact, and chi‐square test to compare the differences between the two groups, as appropriate.

We transformed continuous variables related to laboratory examinations and vital signs on admission into categorical variables. The transformation was based on clinically relevant reference values to facilitate the clinical application of the prediction model. We used CAP as the outcome variable in the database, and the Lasso regression screened the best predictors of the risk of CAP in patients with AEs. The CAP risk prediction model was further developed using multivariate logistic regression. Subsequently, a predictive nomogram was created based on the CAP risk prediction model. The predictive accuracy of the nomogram was assessed by the receiver operating characteristic (ROC) curve and the area under the curve (AUC). Internal validation of the calibration of the nomogram was performed by resampling 500 times using the Bootstrap method. The corrected concordance index and calibration curve were applied to determine the consistency between the actual results and the predicted outcome. We used the decision curve analysis (DCA) to evaluate clinical benefits.

## 3 RESULTS

**FIGURE 1:**
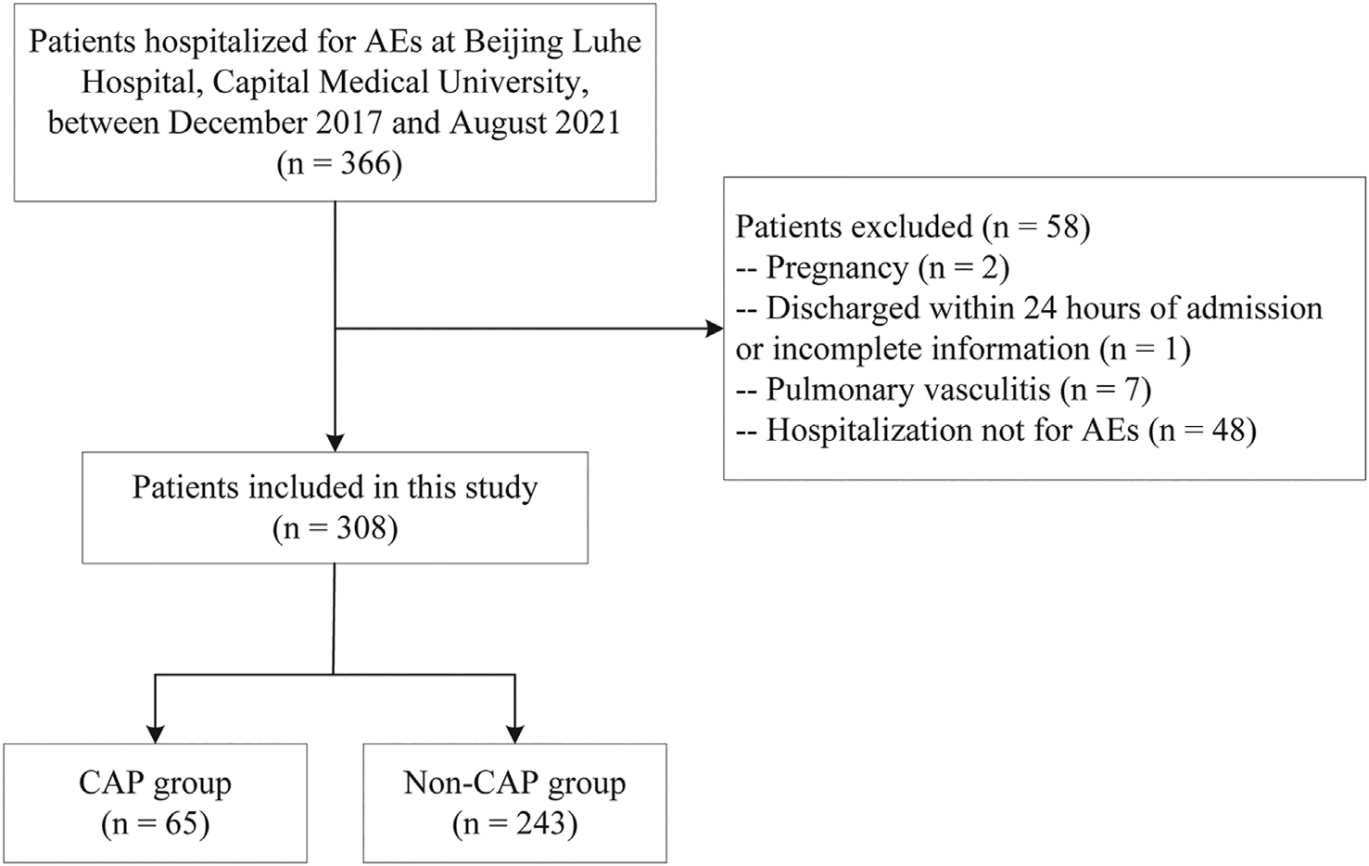
Flow chart of study population selection. AE, asthma exacerbation; CAP, community‐acquired pneumonia.

We screened 366 patients with a discharge diagnosis of AEs between December 2017 and August 2021. Fifty‐eight patients were excluded according to exclusion criteria, and a total of 308 patients were included in the final analysis (Figure 1). The median age of the included patients was 63.00 (52.00, 71.25) years, of which 169 (54.9%) were male, and 139 (45.1%) were female. Chest CT was performed on 304 (98.7%) patients, and chest X‐ray was performed on 4 (1.3%) patients. The patients were divided into a CAP group and a non‐CAP group based on the presence or absence of a CAP diagnosis. There were 243 (79%) patients with a median age of 63.00 (52.00, 72.00) years in the CAP group. The non‐CAP group had 65 (21%) patients with median age of 63.00 (50.00, 70.00) years. The length of stay was 8.00 (7.00, 10.50) and 9.00 (8.00, 12.00) days for the CAP and non‐CAP groups, respectively.

A comparison of the baseline characteristics of the CAP and non‐CAP groups is shown in Table 1. Patients in the CAP group had higher hospital costs (median, 12402.40 vs. 12182.02 Chinese Yuan; *P* = 0.009) and longer asthma duration (median, 7.00 vs. 4.00 years; *P* = 0.027) as compared with the non‐CAP group. Fever, early‐onset asthma, and severe to life‐threatening AEs were more common in the CAP group (*P* < 0.001, *P* = 0.004, *P* = 0.009, respectively). In addition, we found that the CAP group was less likely to use systemic corticosteroid prehospital than the non‐CAP group (26.1% vs. 46.5%, *P* = 0.005). There were no differences observed between the two groups in age, gender, BMI, smoking, family history of asthma, prehospital maintenance medications, history of hospitalization due to AE in the past 12 months, major comorbidities, and the Charlson Comorbidity Index score (*P* > 0.05). In terms of laboratory examinations, patients in the CAP group had higher levels of white blood cells (median, 9.90 vs. 8.06 × 10^9^/L; *P* = 0.001), neutrophils (median, 7.59 vs. 5.37 × 10^9^/L; *P* < 0.001), neutrophil‐to‐lymphocyte ratio (NLR) (median, 5.31 vs. 3.27; *P* < 0.001), platelet‐to‐lymphocyte ratio (PLR) (median, 169.12 vs. 132.26; *P* < 0.001), C‐reactive protein (CRP) (median, 29.01 vs. 4.01 mg/l; *P* < 0.001), and fibrinogen (median, 3.87 vs. 3.18 g/l; *P* < 0.001) as compared with patients in the non‐CAP group. We found no significant difference between the two groups in the peripheral blood eosinophil count and procalcitonin (*P* < 0.05).

**TABLE 1:** Baseline characteristics of the CAP and non‐CAP groups.

Variables	AEs patients without CAP (*n* = 243)	AEs patients with CAP (*n* = 65)	*P*‐value
**Demographic characteristics**			
Age (years old)	63.00 (52.00, 72.00)	63.00 (50.00, 70.00)	0.618
Female	109 (44.9)	30 (46.2)	0.852
BMI (kg/m^2^)	26.38 (23.34, 29.12)	25.22 (22.94, 28.14)	0.135
Smoking			0.078
Nonsmoker	122 (50.2)	32 (49.2)	
Current smoker	67 (27.6)	11 (16.9)	
Former smoker	54 (22.2)	22 (33.8)	
**Clinical outcomes**			
Hospital LOS (days)	8.00 (7.00, 10.50)	9.00 (8.00, 12.00)	0.056
Total costs of hospitalization, CNY	121,82.02 (9656.8114877.22)	145,37.33 (11003.0817987.53)	0.009
**Clinical characteristics**			
Asthma duration (years)	4.00 (0.45, 13.00)	7.00 (2.00, 20.00)	0.027
Early‐onset asthma	77 (31.7)	33 (50.8)	0.004
Family history of asthma	52 (21.4)	21 (32.3)	0.066
Hospitalization due to asthma exacerbation in the past 1 year	45 (18.5)	9 (13.8)	0.379
Asthma exacerbation severity			0.009
Mild to moderate	152 (62.6)	29 (44.6)	
Severe to life‐threatening	91 (37.4)	36 (55.4)	
Prehospital maintenance medications			
ICS	127 (52.3)	35 (53.8)	0.820
OCS	4 (1.6)	0 (0.0)	0.582
ICS types			0.254
Nonusers	114 (46.9)	30 (46.2)	
Budesonide	102 (42.0)	23 (35.4)	
Fluticasone	27 (11.1)	12 (18.5)	
Use systemic corticosteroids before admission	113 (46.5)	17 (26.1)	0.005
**Major comorbidity**			
Hypertension	117 (48.1)	32 (49.2)	0.877
Congestive heart failure	16 (6.6)	6 (9.2)	0.462
CHD	57 (23.5)	12 (18.5)	0.391
Diabetes mellitus	48 (19.8)	11 (16.9)	0.607
COPD	43 (17.7)	16 (24.6)	0.208
Allergic rhinitis	78 (32.1)	22 (33.8)	0.789
Stroke	27 (11.1)	5 (7.7)	0.566
Chronic renal diseases	13 (5.3)	2 (3.1)	0.666
GERD	31 (12.8)	9 (13.8)	0.817
CCI	2 (1, 4)	2 (1, 3)	0.964
**Primary symptoms and vital signs on admission**			
Fever	21 (8.6)	25 (38.5)	<0.001
Respiratory rate (breath/min)	20.00 (20.00., 22.00)	20.00 (20.00, 22.00)	0.604
Heart rate (beat/min)	86.00 (73.50, 97.00)	86.00 (77.00, 101.00)	0.284
Oxygen saturation (%)	96.00 (93.00, 97.00)	96.00 (93.00, 98.00)	0.679
**Laboratory examinations**			
White blood cell (×10^9^/L)	8.06 (6.55, 10.09)	9.90 (7.32, 13.66)	0.001
Neutrophils (×10^9^/L)	5.37 (3.92, 7.32)	7.59 (4.94, 10.37)	<0.001
Neutrophils (%)	66.70 (56.85, 77.25)	77.00 (66.30, 82.70)	<0.001
Eosinophils (×10^9^/L)	0.24 (0.08, 0.60)	0.16 (0.06, 0.41)	0.201
Lymphocytes (×10^9^/L)	1.67 (1.14, 2.24)	1.40 (0.95, 1.85)	0.008
Lymphocytes (%)	20.40 (13.38, 28.50)	13.60 (9.70, 21.40)	<0.001
Platelet (×10^9^/L)	231 (188, 264)	246 (201, 283)	0.101
NLR	3.27 (2.08, 5.60)	5.31 (3.13, 8.20)	<0.001
PLR	132.26 (100.62, 184.66)	169.12 (131.44, 246.59)	<0.001
CRP (mg/L)	4.01 (1.52, 10.14)	29.01 (5.96, 61.20)	<0.001
PCT (μg/L)	0.05 (0.05, 0.06)	0.05 (0.05, 0.07)	0.393
Fibrinogen (g/L)	3.18 (2.75, 3.66)	3.87 (3.36, 4.49)	<0.001
Albumin (g/L)	41.6 (38.9, 44.1)	40.4 (38.4, 42.9)	0.050
BUN (mmol/L)	5.36 (4.06, 6.73)	4.50 (3.78, 6.06)	0.020

Abbreviations: AE, asthma exacerbation; BMI, body mass index; BUN, blood urea nitrogen; CAP, community‐acquired pneumonia; CCI, Charlson Comorbidity Index; CHD, coronary heart disease; CNY, Chinese Yuan; COPD, chronic obstructive pulmonary disease; CRP, C‐reactive protein; GERD, gastroesophageal reflux disease; ICS, inhaled corticosteroid; LOS, length of stay; OCS, oral corticosteroids; NLR, neutrophil‐to‐lymphocyte ratio; PCT, procalcitonin; PLR, platelet‐to‐lymphocyte ratio.

### 3.1 Nomogram development and performance

**FIGURE 2:**
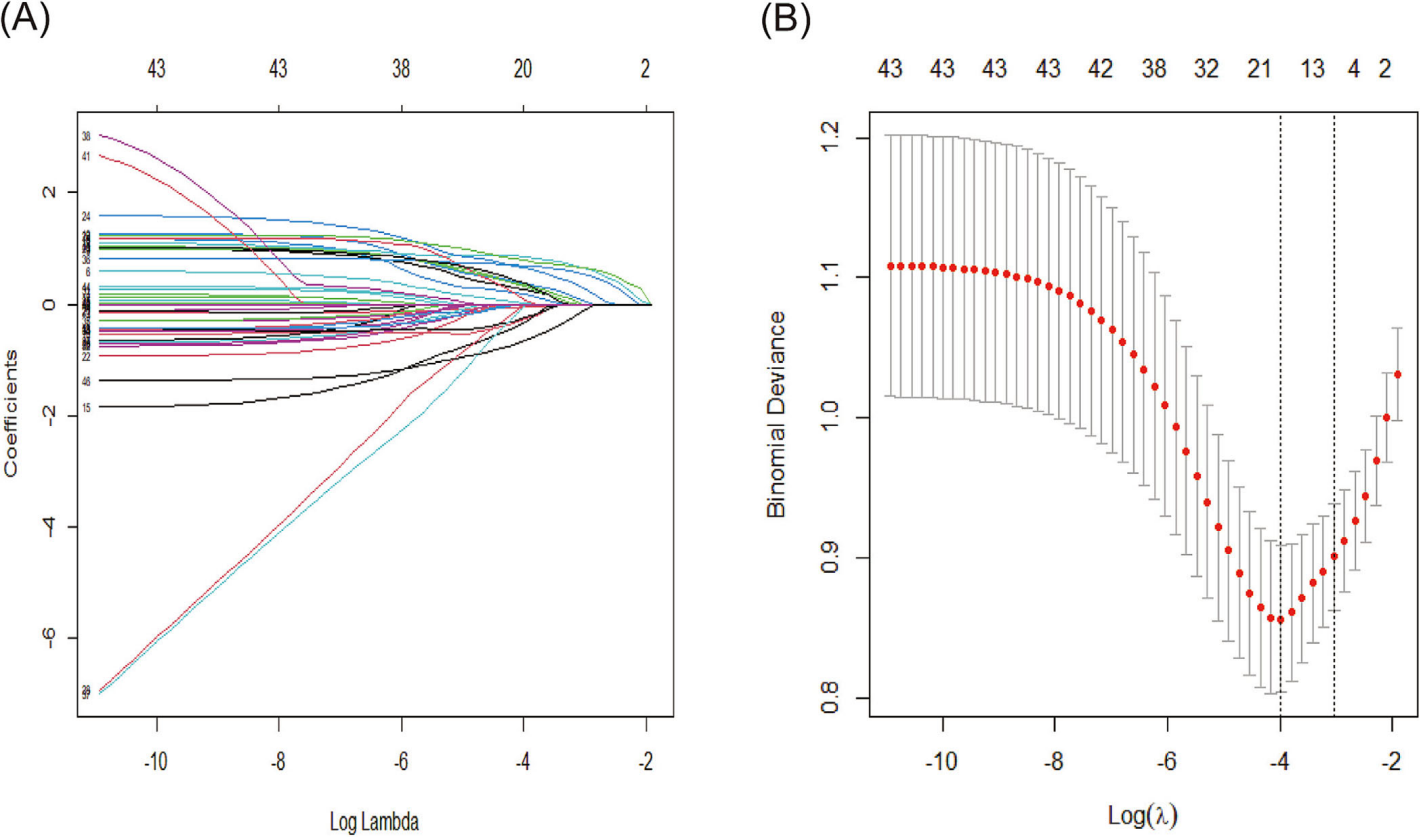
Selection of predictor variables by Lasso regression model. (A) Lasso coefficient profiles of the 54 predictor variables. A coefficient profile plot was produced against the log (λ) sequence. (B) Penalty parameter (lambda) selection in the Lasso model used tenfold cross‐validation via minimum criteria. Dotted vertical lines were drawn at the optimal values using the minimum criteria and the 1 standard error of the minimum criteria (1‐SE criteria).

**FIGURE 3:**
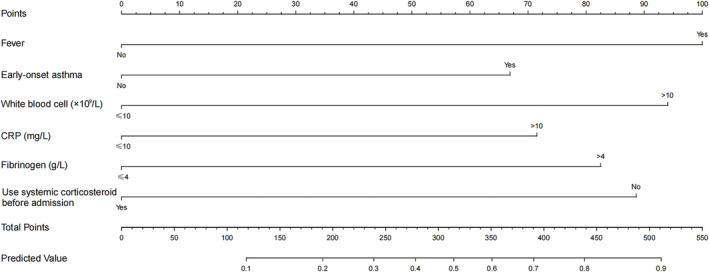
The nomogram to estimate the risk of CAP in adults with AEs. AE, asthma exacerbation; CAP, communty‐acquired pneumonia; CRP, C‐reactive protein.

To facilitate the clinical application of the prediction model, we transformed some of the variables (Table 2). Lasso regression analysis was performed with the occurrence of CAP as the dependent variable and a total of 40 predictor variables as independent variables. The predictors included demographic data, general clinical data, major comorbidities, laboratory examinations, major symptoms, and vital signs on admission. As the penalty parameter λ increased, the selected variables in the model were gradually reduced. The model reached optimal performance when the cross‐validation error was controlled to one standard error of the minimum value. At this point, the corresponding value of λ was 0.048 (Figure 2). A total of five predictor variables were screened, including CRP > 10 mg/L, fibrinogen > 4 g/L, white blood cell >10 × 10^9^/L, fever, use systemic corticosteroids before admission, and early‐onset asthma. These six predictors were included in a multivariate logistic regression analysis, which showed that CRP > 10 mg/L (OR 2.2249, 95% CI: 1.0013–4.8858; *P* = 0.047), fibrinogen > 4 g/L (OR 2.5156, 95% CI: 1.1744–5.3743; *P* = 0.017), white blood cell > 10 × 10^9^/L (OR 2.8620, 95% CI: 1.4695–5.6469; *P* = 0.002), fever (OR 3.0595, 95% CI: 1.3246–7.1749; *P* = 0.009), use systemic corticosteroids before admission (OR 0.3712, 95% CI: 0.1789–0.7390; *P* = 0.006), and early‐onset asthma (OR 2.1125, 95% CI: 1.1031–4.0821; *P* = 0.0245) were all independent predictors of CAP in patients hospitalized with an AE (Table 3). The above six independent predictors were used to develop a predictive nomogram (Figure 3). The individual scores of each risk factor were obtained according to the scale at the top of the nomogram corresponding to that factor. We summed all risk factor scores to get a total score. The probability of CAP occurrence for the patients hospitalized due to AEs was determined based on the total score. A higher total score indicated a higher likelihood of CAP risk. We then plotted the corresponding ROC curves based on the total score (Appendix 1 in the supporting information). According to this curve, we can obtain an AUC of 0.813, a specificity of 81.07%, a sensitivity of 69.23%, and a corresponding cutoff value 207.46. Based on this cutoff value, the total score can be divided into high‐risk and low‐risk groups for subsequent clinical application.

**TABLE 2:** Differences between laboratory examinations and vital signs on admission of CAP and non‐CAP groups after variable transformation.

Variables	AEs patients without CAP (*n* = 243)	AEs patients with CAP (*n* = 65)	*P*‐value
White blood cell > 10 × 10^9^/L	63 (25.9)	32 (49.2)	<0.001
Neutrophils > 70%	100 (41.2)	42 (64.6)	0.001
Neutrophil > 6.3 × 10^9^/L,	92 (37.9)	40 (61.5)	0.001
Eosinophils ≥ 300/μL	117 (48.1)	25 (38.5)	0.164
Lymphocytes < 0.8 × 10^9^/L	23 (9.5)	12 (18.5)	0.042
Lymphocytes < 20%	121 (49.8)	47 (72.3)	0.001
Platelet < 100 × 10^9^/L	6 (2.5)	0 (0.0)	0.349
CRP > 10 mg/L	62 (25.5)	44 (67.7)	<0.001
PCT > 0.1 μg/L	40 (16.5)	14 (21.5)	0.339
NLR > 4	95 (39.1)	42 (64.6)	<0.001
PLR > 198	51 (21.0)	26 (40.0)	0.002
Oxygen saturation < 95%	85 (35.0)	22 (33.8)	0.865
BUN ≥ 7 mmol/L	51 (21)	8 (12.3)	0.114
Albumin < 35 g/L	15 (6.2)	5 (7.7)	0.874
Fibrinogen >4 g/L	35 (14.4)	31 (47.7)	<0.001
Respiratory rate > 20 breath/min	121 (49.8)	28 (43.1)	0.336
Heart rate > 100 beat/min	47 (19.3)	17 (26.2)	0.229

*Note*: We transformed continuous variables into categorical variables based on clinically relevant reference values.

Abbreviations: AE, asthma exacerbation; BUN, blood urea nitrogen; CAP, community‐acquired pneumonia; CRP, C‐reactive protein; NLR, neutrophil‐to‐lymphocyte ratio; PCT, procalcitonin; PLR, platelet‐to‐lymphocyte ratio.

**TABLE 3:** Prediction factors of CAP in patients hospitalized with AEs.

Variables	*β*	Odds ratio	95% CI	*P‐*value
(Intercept)	−2.5267	0.0799	0.0407–0.1458	<0.001
Fever	1.1183	3.0595	1.3246–7.1749	0.009
Early‐onset asthma	0.7479	2.1125	1.1031–4.0821	0.0245
Use systemic corticosteroids before admission	−0.9910	0.3712	0.1789–0.7390	0.006
White blood cell > 10 × 10^9^/L	1.0515	2.8620	1.4695–5.6469	0.002
Fibrinogen > 4 g/L	0.9225	2.5156	1.1744–5.3743	0.017
CRP > 10 mg/L	0.7997	2.2249	1.0013–4.8858	0.047

*Note*: *β* is the regression coefficient. Early‐onset asthma or Late‐onset asthma.

Abbreviations: AE, asthma exacerbation; CAP, community‐acquired pneumonia; CI, confidence interval; CRP, C‐reactive protein.

**FIGURE 4:**
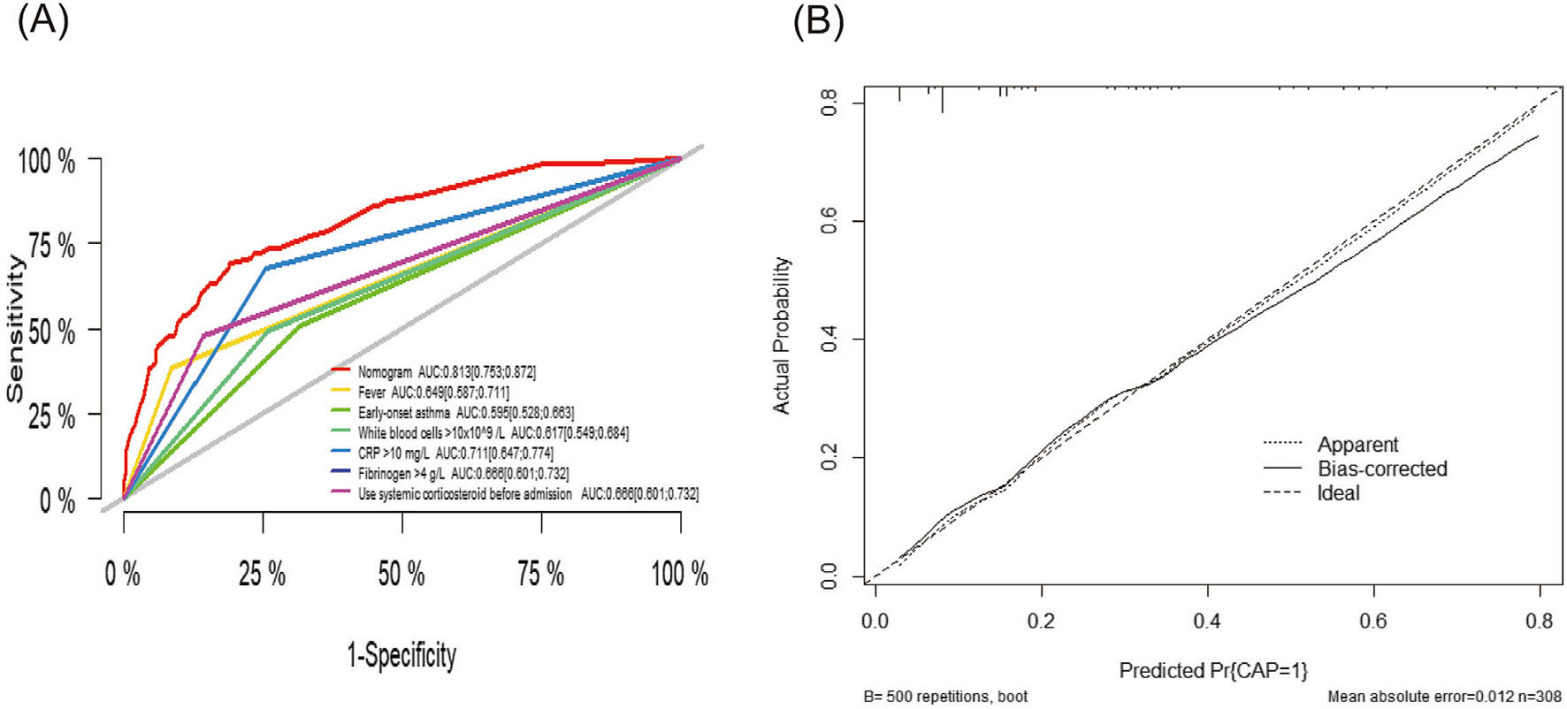
Receiver operating characteristic (ROC) curves and calibration curves of the nomogram. AUC, area under the ROC curve; CAP, community‐acquired pneumonia; CI, confidence interval.

**FIGURE 5:**
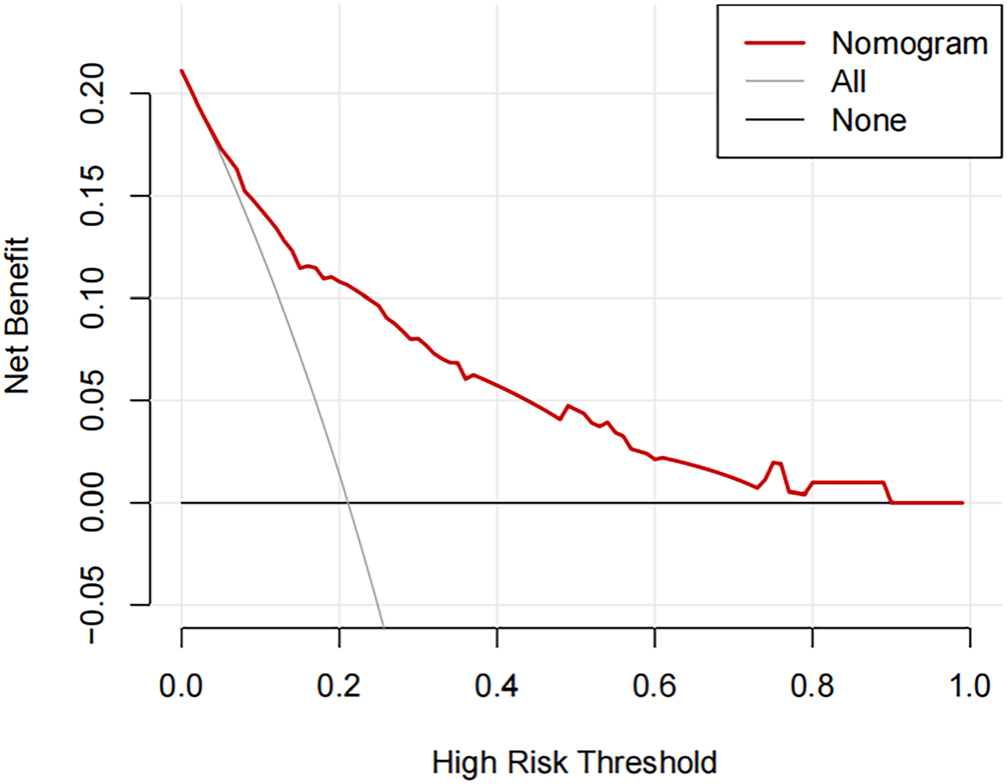
Decision curve analysis (DCA) of the nomogram.

To further evaluate the efficacy of the prediction models, we plotted ROC curves for individual predictor variables and nomogram models (Figure 4). We found a nomogram model AUC value of 0.813 (95% CI: 0.753–0.872), better than each variable. The bootstrap method was used to resample 500 times for internal validation of the nomogram. The corrected concordance index was 0.794. The calibration curve of the nomogram was in high agreement with the diagonal line (Figure 4). The DCA showed that the net benefit of using the nomogram to predict the risk of CAP in hospitalized patients with AEs was high when patients had a threshold probability of 3% to 89% (Figure 5).

### 3.2 Subgroup analysis

We performed a subgroup analysis of early‐onset asthma and late‐onset asthma (Table 4). The results showed that patients in the early‐onset asthma group had a longer duration of asthma (median, 3.00 vs. 20.00 years; *P* = 0.001). In addition, a family history of asthma (*P* = 0.008) and severe acute asthma attacks (*P* = 0.027) were more common in the early‐onset asthma group. The age (median, 66.00 vs. 50.00 years; *P* < 0.001) and comorbidity index (median, 3.00 vs. 1.00 years; *P* < 0.001) were lower in the early‐onset asthma group compared with the late‐onset asthma group. Moreover, the proportion of comorbid hypertension (*P* = 0.002), diabetes mellitus (*P* = 0.022), coronary heart disease (*P* = 0.02), and inhaled corticosteroids (*P* = 0.047) was lower.

**TABLE 4:** Subgroup analysis of early‐onset asthma and late‐onset asthma in patients with AEs.

Variables	Late‐onset asthma (*n* = 198)	Early‐onset asthma (*n* = 110)	*P*‐value
Age (years old)	66.00 (60.00, 73.75)	50.00 (35.25, 67.75)	<0.001
Female	86 (43.4)	53 (48.2)	0.495
BMI (kg/m^2^)	26.26 (23.88, 29.13)	25.36 (22.30, 28.36)	0.061
Smoking			0.063
Nonsmoker	96 (48.5)	58 (52.7)	
Current smoker	57 (28.8)	19 (17.3)	
Former smoker	45 (22.7)	33 (30.0)	
Asthma duration (years)	3.00 (0.25, 10.00)	20.00 (2.00, 40.00)	<0.001
Family history of asthma	37 (18.7)	36 (32.7)	0.008
Hospitalization due to asthma exacerbation in the past 1 year	39 (19.7)	15 (13.6)	0.236
Asthma exacerbation severity			0.027
Mild to moderate	126 (63.6)	55 (50.0)	
Severe to life‐threatening	72 (36.4)	55 (50.0)	
Prehospital maintenance medications			
ICS	113 (57.1)	49 (44.5)	0.047
OCS	2 (1.0)	2 (1.8)	0.94
ICS types			0.065
Nonusers	89 (44.9)	36 (32.7)	
Budesonide	26 (13.1)	13 (11.8)	
Fluticasone	83 (41.9)	61 (55.5)	
Hypertension	109 (55.1)	40 (36.4)	0.002
CHD	53 (26.8)	16 (14.5)	0.02
Diabetes mellitus	46 (23.2)	13 (11.8)	0.022
Allergic rhinitis	59 (29.8)	41 (37.3)	0.224
Stroke	24 (12.1)	8 (7.3)	0.254
Chronic renal diseases	12 (6.1)	3 (2.7)	0.305
CCI	3 (2, 4)	1 (0, 3)	<0.001

Abbreviations: AE, asthma exacerbation; BMI, body mass index; CCI, Charlson Comorbidity Index; CHD, coronary heart disease; ICS, inhaled corticosteroid; OCS, oral corticosteroids.

## 4 DISCUSSION

Respiratory infections commonly cause AEs.^3^, ^22^, ^23^ Some patients with AEs also suffer from CAP. Unfortunately, coincident CAP is associated with asthma‐related adverse outcomes (including endotracheal intubation, cardiopulmonary arrest, and death).^9^, ^10^, ^13^, ^24^ Studies of AEs combined with CAP to date have focused on children.^7^, ^24^, ^25^, ^26^, ^27^ No studies have reported the clinical features of AEs with CAP in adults. Several predictive models of CAP have been developed.^28^, ^29^ However, the prediction models do not focus on the target population of patients hospitalized with AEs. In addition, most of the prediction models were originally for Western patients and may not be appropriate for Chinese patients. We retrospectively analyzed 308 adult patients hospitalized with AEs based on the above status. We found that 21.0% of patients with AEs also had CAP. Using Lasso regression and multivariate logistic regression analysis, we screened out six independent predictors for the occurrence of CAP in adult hospitalized patients with AEs. The six independent predictors included elevated levels of white blood cells, fibrinogen, and CRP, as well as fever, use of systemic corticosteroids before admission, and early‐onset asthma. Subsequently, based on the six optimal predictors, we developed a nomogram to predict the risk of CAP in patients hospitalized with an AE. The nomogram showed good discrimination and calibration capability through internal validation. Based on the DCA, the nomogram demonstrated excellent clinical practicability.

In our study, the CAP incidence in patients hospitalized with AEs was similar to a Korean (20.2%) study,^30^ higher than the studies of Japanese (13.6%)^9^ and Italian (7%)^11^ populations, and lower than that found in Taiwan, China (30%).^13^ In contrast, the incidence of pneumonia in patients admitted to the intensive care unit (ICU) for severe AEs was only 16.8% in a multicenter retrospective cohort study in the United States.^10^ Chest X‐rays are the most commonly used method to diagnose CAP.^31^ However, there is a high rate of underdiagnosis and overdiagnosis of CAP using chest X‐rays. Studies have shown that up to 30% of patients diagnosed with pneumonia on chest X‐ray had no CT findings, whereas up to one third of patients with a negative chest X‐ray had CT imaging consistent with pneumonia.^31^, ^32^, ^33^, ^34^ In Chinese tertiary care hospitals, diagnosis of suspected CAP in patients is mainly based on chest CT.^35^ Chest CT was performed in 98.7% (304/308) of the patients in this study, which reduced the missed diagnosis of CAP. However, none of the previous studies mentioned whether the diagnostic imaging method for pneumonia was based on chest X‐ray or chest CT. We speculated that the significant variation in the incidence of AEs with CAP in different studies might be related to the diagnostic approach to CAP imaging, disease severity, and different geographic regions.

In our study, fever was an independent predictor of CAP in adult patients with AEs. This result was consistent with the findings of studies of pediatric patients with AEs combined with CAP.^7^, ^36^ Increased white blood cells and fibrinogen are common markers for diagnosing CAP. These markers are often used as indicators of the degree of inflammation in the body. In this study, white blood cells and fibrinogen were higher in the CAP group than in the non‐CAP group. Moreover, we found that white blood cells > 10 × 10^9^/L and fibrinogen > 4 g/L were independent predictors of CAP in patients with AEs. These results suggest that the possibility of CAP should be considered for those patients with AEs in the presence of fever and elevated leukocytes and fibrinogen.

CRP is an acute‐phase reactive protein. Proinflammatory cytokines can trigger the synthesis of CRP in the liver. CRP is often used as a biomarker to reflect the degree of inflammation and tissue damage and to evaluate the effectiveness of treatment. Adding CRP to CAP diagnostic models based on the clinical presentation of patients has improved the model's accuracy.^28^, ^29^ One study showed that patients with COPD and CAP had higher levels of CRP than acute exacerbation of COPD (AECOPD).^37^ A similar phenomenon has also been observed in patients with AEs. Our study showed higher CRP levels in patients with AEs and CAP compared with patients without CAP. Moreover, multivariate logistic regression analysis showed that abnormally high CRP level was an independent predictor of CAP in patients with AEs. Wu et al also found a similar result in the study of 120 elderly patients with AEs in China.^38^ Our findings further support the concept that patients with AEs with pneumonia had a more robust inflammatory response. However, as our study was retrospective, most patients' corresponding inflammatory cytokines and induced sputum were not detected. Therefore, it is not clear what type of inflammatory response is involved. However, according to Huang et al., patients with neutrophilic asthma have a more significant airway bacterial load and a more robust inflammatory response than patients with nonneutrophilic asthma.^39^ Therefore, it is possible that the inflammatory response in patients with AEs combined with CAP is likely to be Th1. However, further studies still need to confirm the specific type of inflammatory response.

When an infection occurs in the lungs, alveolar macrophages release cytokines and other inflammatory mediators to eliminate pathogens.^40^ However, an excessive inflammatory response may damage the lungs. Acute application of systemic corticosteroids has been shown to alleviate extreme host inflammatory responses.^41^, ^42^ Our study showed a lower incidence of pneumonia in patients on prehospital systemic corticosteroids. We speculate that this may be related to systemic corticosteroids reducing the systemic inflammatory response.

Asthma is a heterogeneous disease with various clinical phenotypes. Patients can be classified into early‐onset asthma and late‐onset asthma according to their age of asthma onset.^15^, ^16^, ^17^ In our study, we found that the risk of CAP in patients with early‐onset asthma was 1.96 times higher than in patients with late‐onset asthma. A differential study of microbiota in young asthmatics, older asthmatics, and nonasthmatic patients found that some genes were expressed higher in young asthmatics than nonasthmatic patients.^43^ These genes are associated with the pentose phosphate pathway, lipopolysaccharide biosynthesis, flagellar assembly, and bacterial chemotaxis. Researchers believe that the increase in gene expression might be related to increased inflammation and bacterial colonization in young asthmatics. In addition, numerous studies have confirmed abnormalities in the immune defense system of asthma patients.^44^, ^45^, ^46^ Therefore, we postulate that the increased risk of CAP in patients with early‐onset asthma might be related to increased pathogen colonization of the airways. Moreover, studies have demonstrated that prior treatment with ICS reduces the inflammatory response in CAP patients. The potential mechanism might involve the selective regulation of infection defense mechanisms by ICS and inhibition of the classical M1 pathway.^47^, ^48^ This study revealed a low rate of prehospital use of ICS as a controller medication in patients with early‐onset asthma, which could also explain the high incidence of pneumonia in patients with early‐onset asthma in this study.^49^

This study has several strengths. First, in almost all previous clinical asthma studies, patients with a history of heavy smoking or comorbid COPD were usually excluded. However, in clinical practice, many inpatients with asthma have a history of heavy smoking and COPD. Our study included patients with a history of smoking and comorbid COPD, a more realistic clinical scenario. Second, in constructing the prediction model, we did not select predictors based on the results of univariate analysis, as this usually leads to model instability.^50^ We screened the variables using Lasso regression. This method not only achieved the selection of predictor variables but also solved the collinearity problem between variables. However, there are some limitations to our study. First, this was a retrospective study from a single medical center with a case selection bias. Second, external validation was not performed in the prediction model in this study. This might lead to an overestimation of the performance of the nomogram. Third, the diagnosis of CAP was primarily based on imaging, possibly leading to some degree of overdiagnosis.

## 5 CONCLUSION

Adult patients with AEs and CAP had distinct clinical features and occurred with a high incidence. Early‐onset asthma, fever, use of systemic corticosteroids before admission, and some markers (increased levels of white blood cells, fibrinogen, and CRP) were independent predictors of CAP in patients hospitalized for AEs. We developed a simple and reliable nomogram to predict the risk of CAP in patients with AEs. The nomogram had good accuracy, discrimination, and clinical practicability. Using the nomogram, clinicians can determine the risk of CAP in patients hospitalized with an AE. With this prediction model, we can accurately predict the risk of CAP in patients with acute AE and play a role in early screening and diagnosis of CAP. This has an important role in guiding the use of antimicrobial drugs in patients with acute AEs. In summary, the nomogram is a practical and accurate tool for diagnosing and managing CAP in patients with AEs.

## AUTHOR CONTRIBUTIONS

Y. D., J. S., and J. W. designed the study. D. N. and J. Y. contributed to data collection. Y. D., M. L., and Z. H. contributed to the statistical analyses of the study. Y. D. drafted the manuscript. Y. D. and J. W. revised the manuscript. All authors read and approved the final manuscript.

## CONFLICT OF INTEREST STATEMENT

None.

## ETHICS STATEMENT

This study was performed in accordance with the principles of the Declaration of Helsinki. The research was approved by the Ethics Committee of Beijing Luhe Hospital, Capital Medical University (no: 2021‐LHKY‐055‐02). The study was a noninterventional observational study, and the data used were anonymous. Therefore, the ethics committee of Beijing Luhe Hospital, Capital Medical University, issued an informed consent waiver.

## Supporting information


**Data S1.** Supporting Information

**Data S2.** Supporting Information

## Data Availability

Data are available on reasonable request.
