# Supplementary figures and images for: Development of a nomogram to estimate the risk of community‐acquired pneumonia in adults with acute asthma exacerbations
Source: Clin Respir J. 2023 Oct 4;17(11):1169–81. doi: 10.1111/crj.13706 (PMC10632081; doi:10.1111/crj.13706)

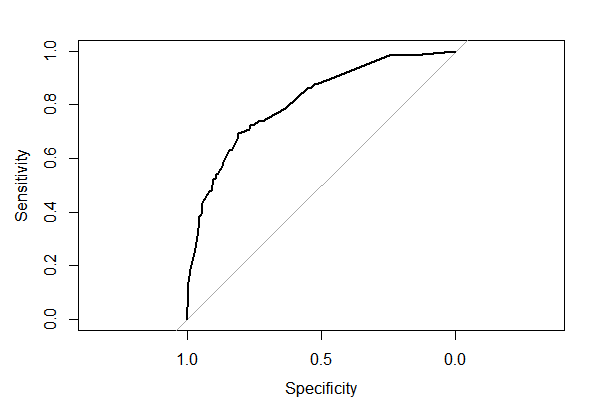

Supplement: Supplementary file 1 — Data S1. Supporting Information [file CRJ-17-1169-s001.png]
